# Biomarkers of pulmonary fibrosis in bronchoalveolar lavage fluid and saliva. Study methods and pathophysiological significance

**DOI:** 10.3389/fmed.2026.1844979

**Published:** 2026-05-25

**Authors:** Cecilia Ángel-Reimúndez, Manuel F. Ruibal-Caballero, Virginia Leiro-Fernández, Milenis Salgado-Arteta, Lucas C. González-Matías, Alberto Fernández-Villar, Federico Mallo

**Affiliations:** 1 Galicia Sur Health Research Institute, SERGAS-UVIGO, Vigo, Spain; 2 Laboratory of Endocrinology, University of Vigo, Vigo, Spain; 3 NeumoVigo I+i Research Group, Galicia Sur Health Research Institute (IIS Galicia Sur), SERGAS-UVIGO, Vigo, Spain; 4 CIBER de Enfermedades Respiratorias, Instituto de Salud Carlos III, Madrid, Spain; 5 Department of Pneumology, Hospital Álvaro Cunqueiro, Vigo, Spain

**Keywords:** BALF (bronchoalveolar lavage fluid), biomarkers, DPLDs, IPF (idiopathic pulmonary fibrosis), KL-6, MMPs (metalloproteinases), saliva, type I collagen

## Abstract

This narrative review synthesizes the current evidence of the molecular biomarkers related to pulmonary fibrosis in bronchoalveolar lavage fluid (BALF) and saliva. It has a particular focus on pulmonary fibrosis diseases (IPFs). It provides a detailed description of the pathophysiological bases and clinical relevance of several extracellular matrix (ECM) components and markers of epithelial damage or remodeling. In this review, it is included type I collagen, hydroxyproline, fibronectin, elastin, KL-6 and matrix metalloproteinases (MMPs). Also, the main methodologies used for their detection and quantification are critically reviewed, highlighting their advantages and limitations in each matrix. Exploring the potential alveolus-saliva pathophysiological axis, it is proposed that this disruption of the alveolar–capillary barrier and the increasing vascular permeability may allow high-molecular-weight biomarkers and matrix derived components to reach the saliva. Thereby, it will enable non-invasive monitoring strategies. However, there are not yet studies that simultaneously analyze BALF and saliva in the same patient cohorts together with the lack of standardized protocol for sampling, analytical normalization and salivary reference ranges. Overall, this review identifies key opportunities and knowledge gaps for the development of BALF-saliva multimarker panels with diagnostic, prognostic and treatment-monitoring potential in fibrotic interstitial lung diseases.

## 1 Introduction

The lungs are the organ most vulnerable to exposure to environmental pollutants, volatile toxins, and microbes potentially promoting tissue damage and having great adverse health effects (1, 2). Lung diseases are a major health problem worldwide and one of the most life-threatening risks to human populations.

There is a large amount of lung diseases classified in various groups like obstructive, occupational or inhalation lung disease, infectious, neoplasia and interstitial-inflammatory diseases. Interstitial lung diseases (ILDs), also referred to as diffuse parenchymal lung diseases (DPLDs), are a very heterogeneous relevant group with increasing incidence in recent decades, characterized by persistent inflammation, fibrosis of the pulmonary interstitium and progressive reduction of lung function with deleterious effects and a much-reduced life expectancy (3). Specifically, idiopathic pulmonary fibrosis (IPF) constitutes a serious and still insufficiently studied complication, making it a highly relevant, infrequent topic of growing interest (4, 5). The diagnosis is based on clinical suspicion, followed by radiological methods of which high resolution computed tomography (HRCT) is central to the diagnosis and classification of ILDs. In addition, for the study of ILD entities, pulmonologist may obtain bronchoalveolar lavage fluid (BALF) from the patient for a more extensive analysis.

BALF extraction is a useful technique that allows to be obtained samples from the distal portion of the pulmonary broncho-alveolar system (6, 7). These samples can be used to isolate microorganisms that can be specifically identified by *in vitro* cultures, stains and PCR (polymerase chain reaction) for strains and serotypes (8–10). This is essential for characterizing and treating resistant infectious lung diseases. In addition, the cell types present in BALF can be detected by flow cytometry analysis, which allows quantifying different inflammatory cells (lymphocytes, eosinophils or neutrophils) and their proportions in IPF diseases and malignant cells in neoplasia (11, 12). In addition, extracellular vesicles (EVs), especially exosomes, may represent a valuable tool for the analysis of BALFs in IPFs, as they carry profibrotic molecules that reflect ongoing tissue damage and contribute to disease progression by acting as active mediators of the disease (13, 14). Finally, the analysis of the composition of the humoral component of BALF allows the detection of molecular markers and pollutant particles, in inflammatory and occupational diseases, respectively.

On the other hand, saliva is becoming increasingly relevant as a biological fluid for the quantification of different biomarkers that can be representative of many different diseases and, therefore, very useful for the diagnosis and monitoring of these diseases (15). It is a biological sample of great interest for clinical applications, as it is easy to obtain and it can be collected repeatedly without risk to patients (16). The quantification of biomarkers in saliva can be carried out using multiple analytical approaches depending on the type of molecule and the clinical goals.

The objective of this narrative review is to synthesize the evidence on molecular markers that can be identified in BALF and saliva and that are relevant for the diagnosis, prognosis, and pathophysiological understanding of lung diseases. In addition, it is interesting to analyze the most commonly used methodologies for detection and characterization of those molecules in both biological fluids as well as the possible correlation between BALF and saliva levels, which is of maximum interest in clinical practice.

## 2 Molecular biomarkers

Molecular biomarkers are molecules such as proteins, nucleic acids, lipids or other metabolites, which help us to understand the relationship between human biology, pathophysiology and diseases. Their presence, lack or alteration in a tissue or biological fluid ease the early detection, diagnosis and treatment of a specific pathology (17).

A useful molecular biomarker must achieve some characteristics: (1) specific, to indicate a particular biological process of a certain disease; (2) perceptible, detecting changes in the early pathological stages; (3) biologically relevant and clinically assessed to show a direct or indirect relationship with the disease, pathogen or physiopathogenic mechanism; and (4) analytical feasibility: biomarkers must be reliably quantified through standardized, reproducible, and clinically accessible methods (18). And for all of the above, thus being useful for medical decision-making.

There are three main types of biomarkers, differentiated according to their clinical utility. First, diagnostic markers demonstrate the presence of a disease or distinguish between pathologies with similar clinical manifestations (differential diagnosis). Second, predictive markers make it possible to assess the progression of the disease and the probability of response to a specific therapy, facilitating personalized treatment. This helps to minimize toxicity and improve the overall prognosis, ensuring a more targeted and effective treatment. Third, prognostic markers assess the severity and expected course of a disease, help estimate the clinical outcome and the specific timelines associated with a given treatment (19, 20).

Despite it is a large amount of molecules with the potential to provide information about IPFs, it is important to focus on those with the greatest likelihood to be implemented in the short term for the evaluation of the disease, as they meet the criteria already conventionally accepted to consider a molecule as a biomarker and are currently available to be measured in BALF and saliva with reliability and reproducibility.

### 2.1 Fibrosis molecular markers

Fibrosis is a pathological state that results from tissue damage followed by incomplete inflammatory resolution that leads to chronification and deposition of fibrotic tissue rather than regeneration of damaged tissue. In this state, the tissue repair mechanism is altered producing excessive amounts of extracellular matrix (ECM).

Although the fibrotic response involves several signaling pathways, the most studied and representative is the transforming growth factor beta-1 (TGF-β1) pathway. When lung tissue is damaged, alveolar epithelial cells and activated macrophages at the site of injury release TGF-β1. TGF-β1 promotes the differentiation of fibroblasts into myofibroblasts. This transition is characterized by the *de novo* expression of α-Smooth Muscle Actin (α-SMA) and the acquisition of a high contractile capacity and strong secretory activity (21, 22). Myofibroblasts are the main cells responsible for ECM deposition; they accumulate at the site of injury forming characteristic agglomerations called fibroblastic foci (23).

This process occurs in connective tissues, where fibrosis puts the interstitium of the lung parenchyma at risk, severely affecting compliance and functionality. In a healthy interstitium there is a physiological balance in the “alveolar-capillary” transition space. The alveolar epithelium and vascular endothelium maintain their integrity. In the interstitium, structure and compliance are preserved. Fibroblasts are scarce and macrophages are inactive (22).

On the other hand, in a fibrotic interstitium the integrity of the alveolar epithelium and vascular endothelium is compromised. Persistent damage and incomplete tissue repair lead to tissue denudation, allowing the infiltration of macrophages, elastin degradation products, and matrix metalloproteinases (MMPs) into the alveolar lumen (23). Type II alveolar epithelial cells (AECII) release Krebs von den Lungen-6 antigen (KL-6), a high molecular weight glycoprotein that subsequently seeps into the systemic circulation under injury-induced stress (24).

**FIGURE 1:**
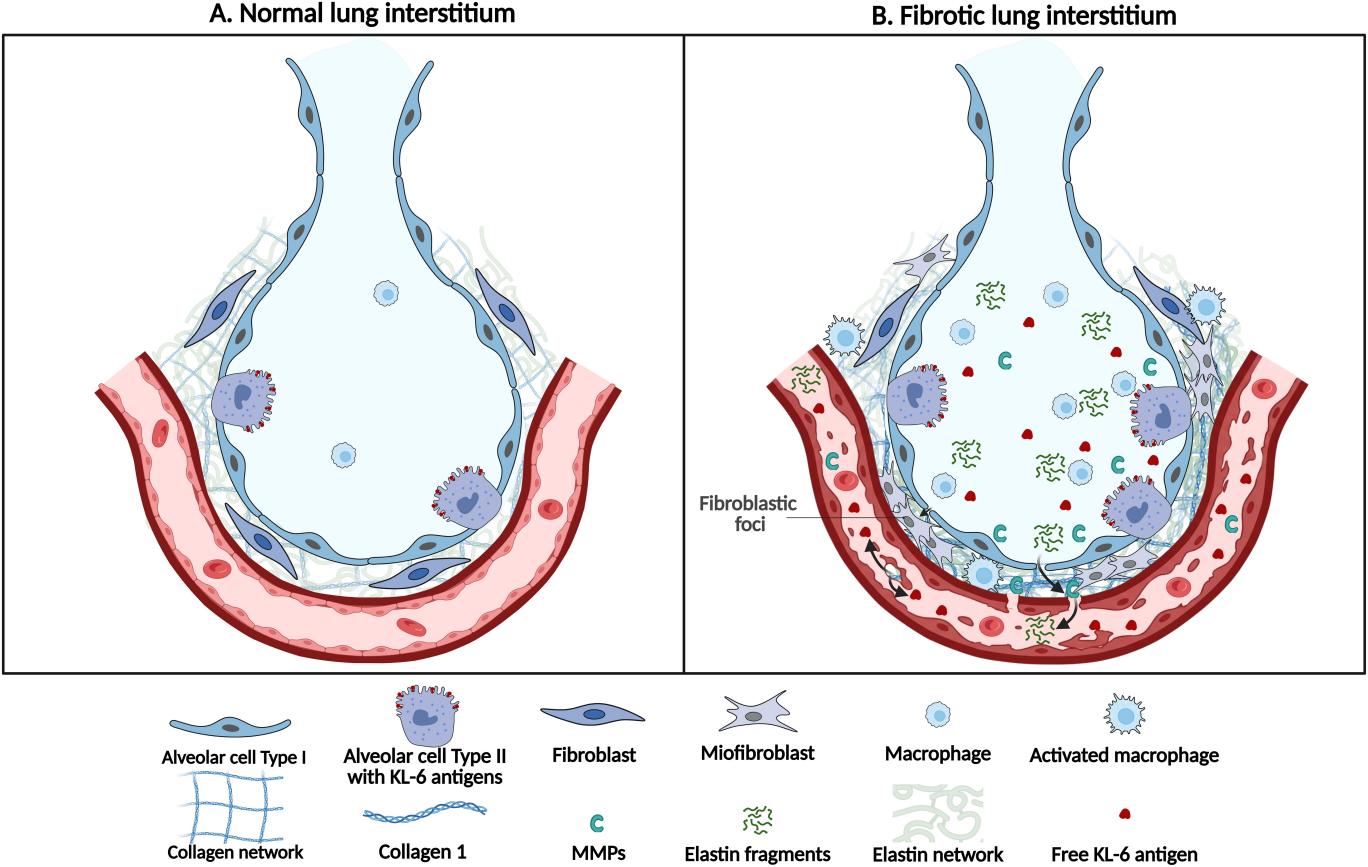
Representation of the most relevant events underlying fibrotic changes in the lung. **(A)** In the healthy lung, the alveoli have intact epithelium and thin interstitial architecture with a balanced presence of collagen, fibronectin and elastin. The alveolar epithelial cells, type I and II pneumocytes have normal function. Type II pneumocytes produces the surfactant lipid and protein components and express the KL-6 (MUC-1) antigen toward the alveolar lumen. **(B)** In the fibrotic lung, the integrity of the alveolar epithelium is altered with excessive deposition of type I collagen **(**COL1**)**, elastin fragmentation, myofibroblast migration and activation and overexpression of metalloproteinases, all of which leads to the general alteration of the extracellular matrix and enlargement of the interstitial space, disturbing oxygen diffusion. The formation of fibroblastic foci promotes tissue destruction, and loss of tissue elasticity, lung compliance and functional deterioration. Created in BioRender by Angel-Reimundez et al. (2026) (https://biorender.com/uxg2iqa).

Myofibroblasts and macrophages migrate to sites of injury to form fibroblastic foci (23). In these foci, myofibroblasts deposit type I structural collagen, fibronectin, which facilitates cell adhesion to foci and MMPs to remodel and replace the “old” matrix with the newly secreted ECM (25). This aberrant deposition of collagen, along with an excessive degradation of elastin, disrupts the physiological balance affecting structure and compliance. An excessive concentration of collagen directly increases interstitial stiffness and, secondly, increases the thickness of the interstitium, thus altering the ability to diffuse respiratory gases (see Figure 1). All of these mechanisms together severely compromise the overall functionality of the lung parenchyma (21, 26).

In this pathological context, the fibrotic molecular markers that we considered to review are those that can be found in elevated concentrations and reflect different stages of the fibrotic process: structural components of the matrix (collagen 1A1, hydroxyproline, fibronectin and elastin), epithelial damage markers (KL-6 antigen) and enzymes involved in remodeling (metalloproteinases).

While individual molecular biomarkers provide valuable insights into specific pathophysiological processes, multimarker panels combining complementary markers of epithelial injury, active fibrosis and matrix remodeling may offer superior diagnostic accuracy and monitoring capacity for IPFs. Especially if both, saliva and BALF, samples are available.

#### 2.1.1 Collagen

Collagen constitutes an essential ECM protein family, whose main function is to provide structural integrity and stability to tissues (27). At least 28 different types of collagens have been identified in humans (28). They are grouped according to their predominant function and localization: fibril-forming, fibril-associated collagens, network-forming and membrane collagens. However, despite having different functions, collagen molecules share a repetitive sequence in each chain: Gly-X-Y, where X and Y correspond to proline and hydroxyproline respectively (29). In type I collagen (COL1), the most abundant isoform in the body, representing approximately 90% of total collagen (30). COL1 has a fibril-forming function, providing mechanical strength and structural support to the ECM, just as type II and type III also have. It is constituted by three polypeptide chains: two α1(I) chains and one α2(I) chain, which are assembled in a triple helix, stabilized by hydrogen bonds (29). 1A1 collagen is the main component of fibers that are deposited in the interstitial space during fibrosis progression.

#### 2.1.2 Hydroxyproline (Hyp)

Hydroxyproline is a non-proteinogenic amino acid that results from a major post-translational modification (PTM) in collagen synthesis that occurs in newly synthesized procollagen chains (31). Hyp is derived from the hydroxylation of proline residues that fills the X and Y positions within the repetitive Gly-X-Y sequence (29).

Proline hydroxylation is mediated by two enzyme families: prolyl 4-hydroxylases (P4H) and prolyl 3-hydroxylases (P3H). The P4H family is responsible for the crucial modification for fibril-forming collagen stability. Specifically, P4H catalyzes 4-hydroxyproline formation at the Y locus (31). This 4-hydroxylation provides stability to the triple helix (32). The hydroxyl group (−OH) has a high electronegativity and induces specific conformation in the proline ring, which predisposes the peptide chain of the geometry to assemble the triple helix [Gauche effect, (31)]. In addition, this −OH network promotes stability by water-mediated hydrogen bonds, providing favorable stabilization for collagen molecules (33).

#### 2.1.3 Fibronectin

Fibronectin is an abundant ECM glycoprotein necessary for cell adhesion, migration, differentiation and tissue repair. This molecule provides a scaffold structure for other protein assemblies in the ECM, principally collagens to contribute to the structural integrity of the pulmonary parenchyma (25). In adult lungs, its main function is associated with repair processes after tissue injury guiding the migration of pro-inflammatory cells and fibroblasts to the injured areas.

There are several fibronectin isoforms generating by alternative splicing. One of them is the extra A domain (EDA) isoform, which is of great importance because it promotes inflammatory signaling pathways that contribute to the pathogenesis of organ fibrosis (34, 35). Moreover, it plays an important role in transforming fibroblasts into myofibroblasts, the cells responsible for excessive collagen production in the ECM during fibrosis.

Fibronectin expression can be induced by proinflammatory cytokines such as TGF-β, interleukin-1 beta (IL-1β), and tumor necrosis factor-alpha (TNF-α) and by growth factors, such as platelet-derived growth factor (PDGF) and fibroblast growth factor (FGF) (25, 34–38). Instead, fibronectin degradation depends on metalloproteinase activity, which modulates tissue remodeling during fibrosis and ECM fixation. The imbalance between the synthesis and degradation of fibronectin leads to the development of different diseases (39, 40).

#### 2.1.4 Elastin

Elastin is another essential structural protein in connective tissue which provides elasticity to organs, mainly those that expand and contract repeatedly. In the lungs, elastin is part of the ECM by composing a three-dimensional network within the lung parenchyma, along with collagen and other proteins (41). Thanks to this network, alveoli can expand during inspiration and regain their size during expiration, ensuring compliance and mechanical efficiency of respiration. Its degradation or assembly alterations cause loss of elasticity and reduced pulmonary compliance in patients with emphysema and chronic obstructive pulmonary disease (COPD) (42).

Elastin is produced from tropoelastin, which is mainly synthetized during the perinatal period. Tropoelastin monomers are intertwined by the action of the enzyme lysyl oxidase to form strong elastin fibers. This process generates amino acids unique to elastin: desmosine and isodesmosine, which are only found in insoluble elastin (16) and requires a delicate balance between elastin production and degradation. Elastin production is higher in developing lungs and decreases with age, making it difficult for damaged lungs to regenerate throughout life. Due to the low rate of elastin turnover, the presence of protein fragments or the specific amino acids desmosine and isodesmosine in serum, urine, sputum or BALF is a useful biomarker of elastin degradation by inflammation-associated elastases. Specifically, the levels of sputum/BALF desmosine and isodesmosine reflect the degree of elastin degradation in the lung (26, 43, 44).

For several years, neutrophils were believed to be the cells responsible for tissue destruction in emphysema by the release of elastase. Elastase expression and its enzymatic activity were associated with COPD (45). Currently, it is known that the degradation of elastin depends on proteolytic enzymes such as neutrophil elastases, but also elastases produced by macrophages, metalloproteinases and cathepsins, which are regulated by endogenous inhibitors such as α1-antitrypsin. If the balance between these enzymes were to be disturbed, irreversible damage to the lung parenchyma could occur (46–48).

#### 2.1.5 KL-6

KL-6 is a specific epitope of mucin 1 (MUC1). MUC1 is a transmembrane glycoprotein expressed mainly by AECII and to a lesser extent by bronchial epithelial cells. This protein has three domains: cytosolic, transmembrane, and extracellular (47, 48).

KL-6 is located in the ectodomain of MUC1 (49). At present, the concentration of soluble KL-6 in the serum is regarded as a biomarker directly proportional to the degree of alveolar epithelial damage and/or regeneration (24). Although the mechanism by which it is released from the membrane has not been fully clarified, it is accepted that it results from a combination of complementary pathways. Lillehoj et al. (50) identified the desialylation of the ectodomain by neuraminidase 1 (NEU1), removing terminal sialic acid residues, exposing a protease-sensitive region and thus facilitating the action of cleavage proteases called sheddases (50). On this proteolytic action some sheddases such as matrix metalloproteinase-14 (MMP-14) have an important role, as well as the γ-secretase complex (48). Recently, Zhang et al. (24) proposed that the MUC1 ectodomain (thus KL-6 antigen) is fixed on the surface of AECII by intermolecular disulfide bonds. During epithelial injury, the breaking of these bonds allows for the abrupt release of soluble forms of KL-6 into the alveolar lumen (24).

#### 2.1.6 Metalloproteinases

Matrix metalloproteinases are zinc-dependent endopeptidases that play a very important role in the degradation and remodeling of ECM components. They are produced by various pulmonary cell types, such as epithelial and endothelial cells, fibroblasts and alveolar macrophages (51, 52). It has been found that MMP activity is regulated by specific inhibitors called tissue inhibitors of metalloproteinases (TIMPs) (52, 53). These bind to MMPs in a non-covalent manner, forming a reversible and high-affinity complex when MMPs are structured as proenzymes.

In the absence of pathology, MMPs levels are usually low in adult tissues. This is important to maintain the balance between matrix synthesis and degradation. However, when MMP expression increases, the integrity of the epithelial barrier is altered, promoting the migration of inflammatory cells into the pulmonary interstitium and intensifying an abnormal repair response. This generates a vicious cycle of inflammation, remodeling and fibrosis, which leads to progressive stiffening of the lung parenchyma and irreversible deterioration of respiratory function (39).

#### 2.1.7 Emerging biomarkers

Recent multi-omics studies have identified novel biomarker candidates that reflect specific cellular states in IPF, complementing established ECM and epithelial damage markers. These emerging molecules, detected in BALF, plasma or serum, need further validation for clinical translation, particularly in non-invasive fluids like saliva. This anticipates the possibility that expanded biomarker panels for IPFs will appear in the near future, of which an updated example is shown below.

Periostin: This extracellular matrix protein may act as a chemokine inducer playing a role in the recruitment of neutrophils and macrophages during pulmonary fibrosis in mice models and human. Recent evidence also highlights its clinical relevance as a biomarker for the diagnosis and assessment of the degree of fibrotic involvement in specific IPFs (54). It is elevated in the blood of IPF patients and good correlation with KL-6 and fibrosis score on HRCT (55). It is also elevated in other frequent lung pathology as asthma, lung cancer, eosinophilic pneumonia and chronic respiratory diseases (56, 57). It has been identified in saliva related to periodontitis (58), as highly expressed in the periodontal ligament (59). Periodontitis has a prevalence of 62% in the general population (60). All those are confounding factor that may undermine the usefulness of periostin in the detection and monitoring of interstitial lung diseases.

CFHR1 (complement factor H-related protein 1) and CRTAC1 (cartilage acidic protein 1): CFHR1 and CRTAC1 are molecules emerged from integrative single-cell RNA-seq and plasma proteomics analyses as peripheral indicators of pericyte SSTR2+ activation and alveolar type 2 cell health, respectively. Both were found to be elevated in the BALF and plasma of IPF, correlating with disease severity and providing information on fibrotic cell transition (61, 62). Not still found in saliva.

CX3CL1 (fractalkine): This is a chemokine involved in the recruitment of macrophages and the activation of fibroblasts, which has high levels in the serum and bronchoalveolar lavage fluid of patients with IPF, which could link inflammation with the remodeling of the extracellular matrix, although its value meaning requires studies with large cohorts (63). Found in saliva may be altered in other lung diseases as tuberculosis, inflammatory diseases and cancer (64, 65).

CD44: CD44 is a hyaluronan receptor in exosomes, which was recently validated in bronchoalveolar lavage fluid as a discriminatory marker for diffuse parenchymal lung diseases, including idiopathic pulmonary fibrosis, indicative of epithelial-mesenchymal transition processes (66). Found in saliva, may be largely affected by oral pathology (67, 68).

## 3 Quantification in BALF and saliva

**FIGURE 2:**
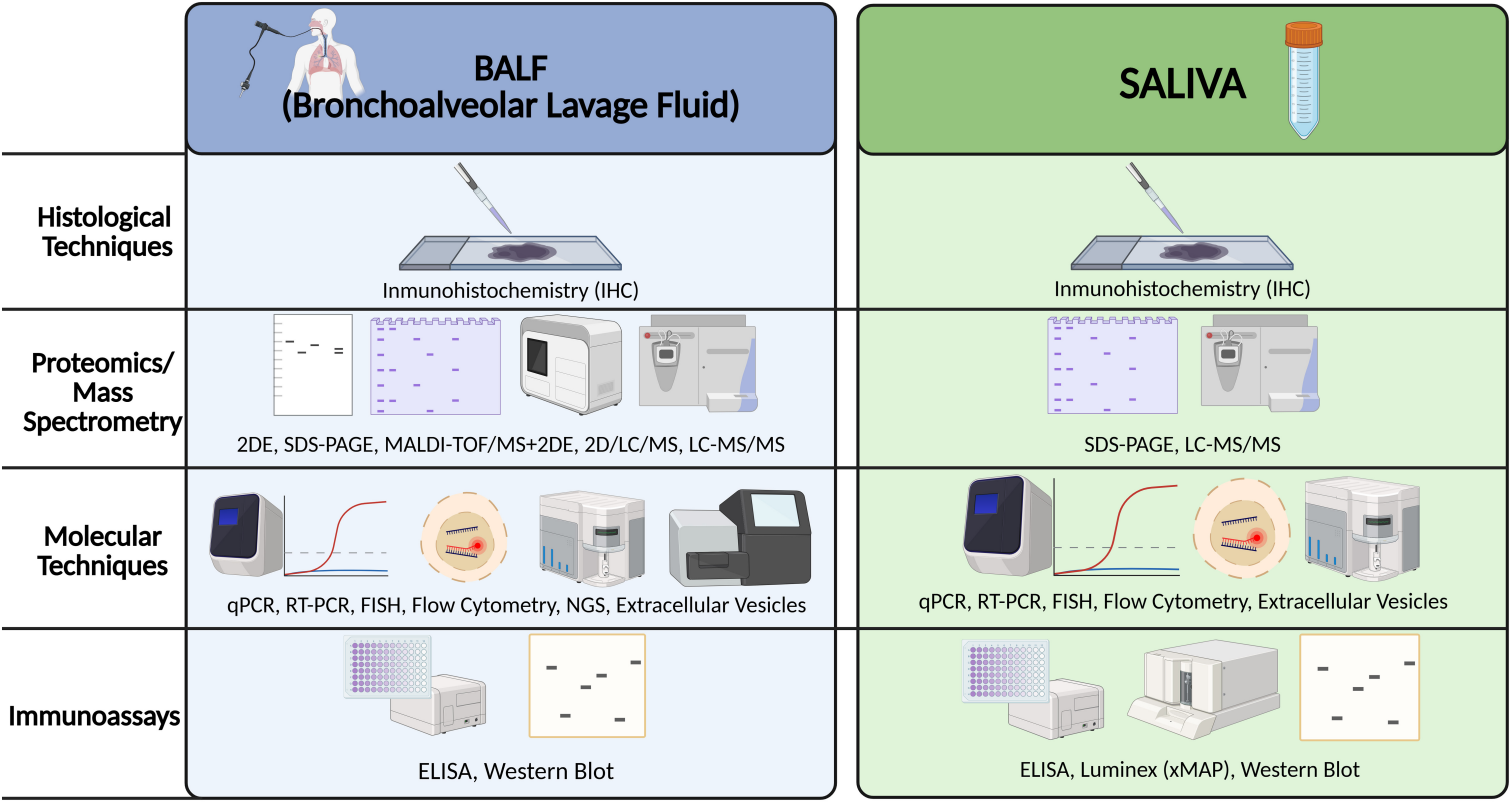
Summary of the methodologies used to detect biomolecules in BAL fluid and saliva. Created in BioRender by Angel-Reimundez et al. (2026) (https://BioRender.com/30nj5nv).

In the respiratory disease context, BALF is the most representative biological sample to identify molecular biomarkers that reflect the cellular and molecular changes in respiratory airways. There are several ways to quantify molecular biomarkers in BALF for the detection or assessment of different lung diseases. Similarly, saliva can also be used to detect certain molecules derived from metabolism, pulmonary inflammatory or tumor processes, such as proteins, microRNAs, metabolites or circulating free DNA. To achieve this, various methodologies may be employed, as: histological techniques, proteomic and mass spectrometry techniques, molecular biology methods and immunoassays (see Figure 2).

### 3.1 Histological and cellular techniques

BALF contains different cell types representing alveolar-bronquial populations that can be revealed by histological techniques using conventional staining. Cytospin-stained BALF preparations typically show a mixed cell population on a lightly stained background of protein material and mucus (69). Alveolar macrophages are the predominant cell type, appearing large with abundant cytoplasm often vacuolated and round to oval nuclei (70); some may contain ingested particles or pigments. Lymphocytes are identified as smaller cells with scanning cytoplasm and dense, round nuclei. Neutrophils and eosinophils characterized by segmented nuclei and granular cytoplasm may occasionally be observed, depending on the inflammatory process (71). Epithelial cells can be seen sporadically, appearing to be larger, with more cytoplasm and fewer condensed nuclei. In general, cellularity and differential cellular composition of BALF vary according to the clinical context and the underlying disease.

A methodology used in a complementary manner to other techniques is immunohistochemistry (IHC). Although it has traditionally been associated with the analysis of tissue samples, recent studies have adapted similar principles for application to cells present in saliva and BALF through approaches based on “liquid cytology” (72, 73). IHC does not allow for quantification of biomarkers but rather enables the detection and spatial localization of molecules within the anatomy of lung tissues, specially the respiratory epithelium. In the study of interstitial, neoplastic or infectious diseases, it is commonly applied to transbronchial or surgical lung biopsies. However, in some cases it is also applied to cytological material generated from BALF. In this case, immunostaining with antibodies against lymphocyte, macrophage, epithelial cell or infectious agent markers, allows the validation and correlation of molecular findings with cellular characteristics (23, 25, 51, 52, 74).

#### 3.1.1 Fluorescent *in situ* hybridization (FISH)

FISH with break-apart probes has been mainly applied to detect gene rearrangements in cells present in respiratory samples, especially in non-small cell lung cancer (75, 76). Unlike RT-PCR and NGS, FISH is performed *in situ* on fixed cells without prior DNA extraction, which allows maintaining the morphological context and combining molecular information with cellular localization.

Likewise, the FISH technique has been applied to saliva samples to detect and locate nucleic acid sequences directly in cells present in the fluid using oligonucleotides labeled with fluorochromes. Currently, FISH protocols in saliva are described mainly for the detection of pathogen RNA or DNA with sensitivities and specificities comparable to RT-qPCR, demonstrating the viability of saliva as a matrix for *in situ* hybridization (77, 78).

#### 3.1.2 Flow cytometry

Flow cytometry is a fundamental tool for the analysis of BALF. It allows immunophenotyping of different cell subpopulations present in BALF, such as lymphocytes, monocytes, and neutrophils (12). To achieve this, fluorescent antibody panels targeting markers such as CD3, CD4, or CD8 are used (79–81). This helps establish cellular patterns typical of certain diseases such as sarcoidosis, hypersensitivity pneumonitis or drug-induced pulmonary toxicity (46).

Flow cytometry can be used to quantify cell subpopulations and analyze extracellular vesicles or microparticles marked with specific antibodies in saliva samples. This provides information (82, 83) relevant to identifying cellular phenotypes and expression of surface proteins associated with inflammatory processes or tissue remodeling in lung diseases (84, 85).

Flow cytometry is also increasingly used for phenotyping of extracellular vesicles, including exosome-enriched fractions, by analyzing surface markers and particle-associated proteins, which may support the detection of disease-related EV signatures in BALF and other biological fluids, such as saliva (86).

### 3.2 Proteomic and mass spectrometry techniques

Proteomic techniques have been widely applied in numerous studies on BALF and saliva as tools to characterize biological microenvironments and identify diagnostic or prognostic biomarkers of different respiratory diseases. In the case of BALF, this approach allows defining patterns of protein expression that may be associated with inflammatory, infectious, neoplastic or fibrotic processes, since it contains a complex mixture of proteins derived from the alveolar epithelium, immune cells and interstitial fluids (87, 88).

Among the classical methodologies used, two-dimensional gel electrophoresis (2DE) stands out because it allows the simultaneous separation of hundreds of proteins according to their isoelectric point and molecular mass. This technique is particularly relevant in BALF because it prevents the comigration of epithelial, immune and serum proteins present in this fluid, which occurs with one-dimensional techniques such as SDS-PAGE. Moreover, 2DE enables the detection of subtle changes in protein expression or post-translational modifications associated with disease, and comparison of proteomic profiles between control groups and different pulmonary pathologies (89). This technique is especially useful when combined with MALDI-TOF/MS mass spectrometry, which enables the identification of specific proteins or peptides present in BALF (6, 90, 91).

The development of high-throughput proteomics platforms such as two-dimensional liquid chromatography coupled to mass spectrometry (2D-LC/MS) and liquid chromatography platforms coupled to tandem mass spectrometry (LC-MS/MS), reduces the complexity of the mixture entering the analyzer and increases the likelihood of detecting relevant but scarce proteins as biomarkers of inflammation, infection or neoplasia (87). These approaches have been used to compare BALF proteomic profiles between healthy subjects and patients with COPD, asthma, pulmonary fibrosis, pneumonia and suspected lung cancer. Important differences in proteins related to immune responses, matrix remodeling and oxidative stress were identified by these methods. In current practice, the high-throughput LC-MS/MS is replacing the 2DE in discovery studies (90, 92).

Similarly, proteomic approaches in saliva allow characterizing the salivary proteome with the advantage of using an accessible and non-invasive sample (93). Recent studies use LC-MS/MS techniques with isotopic labeling to detect proteins of low abundance and their relative proportion in saliva. In this way, it is possible to construct proteomic profiles associated with disease states, lung function or fibrotic progression (94). In this context, proteomic studies in saliva have identified panels of candidate proteins that are subsequently validated by targeted immunological assays (95, 96).

In addition, proteomic workflows can also be applied to exosome-enriched fractions, enabling the characterization of vesicle-associated proteins with potential diagnostic value in fibrotic lung diseases (97).

### 3.3 Molecular biology methods

Among the most commonly used techniques are quantitative PCR (qPCR), real-time PCR (RT-PCR), and next-generation sequencing (NGS).

Several biomarkers can be detected at the nucleic acid level in the BALF. Molecular techniques allow the detection and quantification of genetic material of both pathogens and hosts, with high sensitivity and specificity, even when the cellular or microbial load is very low. Nucleic acid amplification can identify opportunistic infections, respiratory viruses or mycobacteria, as well as genetic alterations related to cancer or other lung diseases, from a small volume of BALF.

In addition, these methodologies allow the molecular characterization of diseases including the biological pathways and inflammatory mechanisms involved in pulmonary pathophysiology.

Beyond free nucleic acids and soluble proteins, extracellular vesicles, particularly exosomes, represent an additional molecular compartment with relevant diagnostic and pathophysiological information in BALF and saliva.

#### 3.3.1 qPCR and RT-PCR

qPCR and RT-qPCR have been used in numerous studies for the detection of respiratory viruses, such as SARS-CoV-2 or influenza, fungal infections and pneumonias, as well as in the diagnosis of tuberculosis and mycobacterial infections, or the identification of genes involved in the pathophysiology of the disease, the immune, inflammatory and lung tissue response (8, 9, 98–100). This technique is capable of detecting the expression of almost any specific mRNA species under validated conditions. In recent studies in patients with acute exacerbations of COPD and associated pneumonia, RT-PCR in BALF has made it possible to quantify the mRNA expression of pro-inflammatory cytokines such as interleukin-6 (IL-6), interleukin-8 (IL-8) and TNF-α and it has been shown that the levels of these mediators in BALF decrease after therapeutic interventions (101). Similarly, in cohorts of smokers and COPD patients, RT-PCR in BALF has been used to identify gene expression profiles linked to oxidative stress and lower respiratory tract inflammation (102, 103).

At the same time, qPCR in saliva samples has made it possible to expand the analytical spectrum from proteins to transcriptomic or genetic signatures. After RNA extraction and reverse transcription, real-time amplification allows for sensitive and reproducible quantification of many mRNA species, if rigorous control of RNA quality and correct normalization of results are ensured (104). This technique has been used in studies on asthma, COPD and other respiratory diseases (105–107), as well as to assess viral load and therapeutic sensitivity in acute lung infections (108, 109).

In addition, massive sequencing has expanded molecular diagnostic capabilities in bronchoalveolar lavage fluid (BALF) in infections of unknown etiology or in immunocompromised patients (10, 110, 111). Also in oncology, cell-free tumor DNA or tumor cells present in BAL can be used for specific panels of point mutations, insertions or deletions, and rearrangements in target genes (112, 113).

#### 3.3.2 Extracellular vesicles and exosomes

Extracellular vesicles have emerged as promising analytes in BALF and other biofluids because they transport proteins, lipids and nucleic acids that reflect the molecular state of the lung microenvironment (13, 14). These membrane-surrounded particles released by different cell types in lung tissue, such as pneumocytes, fibroblasts and macrophages, contain specific factors that modulate fibrogenic signaling and inflammatory response (114, 115). Several studies have shown that exosomal or EV-associated microRNAs in IPF, including miR-21 and miR-199a-5p, are related to profibrotic signaling and fibroblast-myofibroblast differentiation (116, 117). These findings support the idea that exosomes can act both as biomarkers and as active mediators of fibrotic progression. Overall, current evidence positions exosomes not only as potential diagnostic tools, but also as active contributors to fibrotic progression, opening up new perspectives for the development of therapeutic strategies aimed at modulating their release or loading (118).

### 3.4 Immunoassays

Immunoassays are very efficient techniques combining high sensitivity and high specificity to detect molecules of interest in biological liquids. They allow the measurement of specific soluble proteins such as cytokines, chemokines, MMPs or specific antigens and antibodies. These tests have demonstrated usefulness in the early diagnosis and monitoring of respiratory and infectious diseases through colorimetric or chemiluminescent readouts. Many studies have used specific immunoassays in BALF to analyze changes in the components of the pulmonary extracellular matrix (37, 46, 119).

One of the most commonly used immunoassays for the quantification of biomarkers in BALF and saliva is the ELISA-type immunoassay. These are the most widely employed formats due to the availability of commercial kits. Plate ELISAs have been widely used in BALF to quantify various biomolecules and characterize the immune microenvironment and the degree of lung damage. Among the most studied are pro-inflammatory and anti-inflammatory cytokines such as IL-1β, IL-6, IL-8, TNF-α, IL-10, and TGF-β; lung-specific proteins such as surfactant proteins; immunoglobulins; and markers of permeability and tissue damage such as albumin (120, 121). And in saliva to quantify biomarkers such as C-reactive protein, procalcitonin, neutrophil elastase, or alpha-1 antitrypsin (122, 123), which allow assessment of their diagnostic potential in diseases such as asthma or COPD.

In addition, multiplex platforms have been developed, such as systems based on color-coded microspheres (Luminex/xMAP), which allow the simultaneous detection of several proteins in the same sample volume. This approach has made it possible to study salivary biomarkers such as lactoferrin, IL-8 or MMPs associated with inflammatory processes characteristic of COPD, pulmonary fibrosis or lung cancer (124).

Western blotting, in turn, although less suitable for high-throughput analysis, it is useful when, in addition to the quantity, the size, isoform or integrity of a particular protein is of interest. This technique allows proteins to be separated by transferring them to a membrane containing specific antibodies for detection. When it is combined with sodium dodecyl sulfate–polyacrylamide gel electrophoresis (SDS-PAGE) and immunohistochemical detection, it enables confirmation of the presence, molecular weight, and modifications of proteins of interest. Western blotting is used for the analysis of BALF and saliva because it allows differentiating between protein subtypes, verifying post-translational modifications and validating results obtained by other techniques such as ELISA or PCR (34, 74, 125).

## 4 Predictive value of biomarkers in BALF and saliva

BALF is obtained through bronchoalveolar lavage, a routine procedure that requires preoperative preparation and patient sedation. Therefore, the nature of the technique itself is highly invasive and not absent of same risk of complications This procedure can be uncomfortable and may even cause anxiety or fear in patients prior to its performance. However, BALF is a high value diagnostic tool (7). The composition of BALF is abundant in cells and cellular components, proteins, metabolites, biomarkers, and even pathogenic microorganisms present in the distal respiratory tract and the alveolar epithelium (69). Saliva, on the other hand, represents a particularly noteworthy alternative sample obtained in a non-invasive procedure, without risk of complications in conscious subjects and highly repeatable. However, the potential of saliva to provide information about any pulmonary pathophysiological event must be rigorously demonstrated, as salivary composition can be affected by various conditions, including local (oral) factors, secretory ratio, metabolic status and digestive diseases. Therefore, changes in saliva may not solely reflect pulmonary physiology or disease.

Saliva is a dense and viscoelastic biological fluid rich in biomolecules containing both organic molecules (e.g., enzymes) and inorganic molecules (e.g., metal ions). The salivary proteome comprises around 2,000 proteins and peptides, 27% of which are also present in plasma, having potential in the diagnosis, monitoring and prognosis of diseases (126). In recent years, saliva has become increasingly relevant in translational medicine as a fluid of interest due to its minimally invasive collection and the possibility of daily sampling without discomfort for disease monitoring. Both of these reasons make saliva a potential option for early diagnosis and for the development of point-of-care (POC) diagnostic tests, as was the case with COVID-19 (127). According to Pittman et al. (127), it is potentially useful in the management of systemic diseases such as diabetes, head and neck, oral, breast and lung cancer, heart failure, periodontal diseases and drug abuse (128–135).

All the biomarkers studied in this work have previously been shown to be of interest as pulmonary biomarkers and are therefore reviewed here to identify their involvement in certain lung diseases with special emphasis on interstitial lung diseases.

### 4.1 Relationship between BALF and saliva values

The detection of high–molecular-weight and extracellular matrix biomarkers in fluids outside the alveolus can be explained by the loss of integrity of the alveolar–capillary barrier. This is a pathological event common in fibrotic-type IPFs. Alveolar epithelial injury and basement membrane disruption increase vascular permeability, allowing molecules, such as KL-6, collagen and elastin degradation products, to enter systemic circulation. These molecules subsequently reach salivary glands through blood ultrafiltration mechanisms for producing the saliva (136, 137). Recent reviews confirm that damage to the basement membrane and the consequent increase in vascular permeability allow the passage of macromolecules from the alveolus into the bloodstream. Hypothetically, once these molecules enter the circulation, they can be secreted in saliva, and saliva values can be representative of the disease (24). The clinical interpretation of salivary biomarkers, compared to BALF, would depend on their context within the spectrum of IPF disease and other common interfering diseases, especially those affecting the oral cavity, as has already been demonstrated for some of the potential emerging biomarkers mentioned above, and which will require specific studies to be developed.

#### 4.1.1 Markers of active fibrosis: COL1, Hyp, fibronectin, elastin

COL1, Hyp, fibronectin, elastin, and MMPs are ubiquitously distributed molecules in connective tissues, but not KL-6, which is expressed in glandular epithelia such as lung tissue and salivary glands (47, 138). During fibrosis, fibroblasts and myofibroblasts secrete high concentrations of COL1 and fibronectin, among other proteins, to counteract the loss of connective tissue integrity, grouping together in secretion sites known as fibroblastic foci (23). To secrete COL1, myofibroblasts overexpress the COL1A1 and COL1A2 genes, which determines the start of the fibrotic process, while excessive tissue concentrations of COL1 protein reflect more advanced or persistent stages over time.

The functional molecules of COL1 require a parallel increase in P4H enzyme activity to enable the post-translational hydroxylation of proline residues to Hyp. During collagen degradation, specific degradation products can be released into the extracellular environment. Hyp is an amino acid unique to collagen molecules and is directly associated with *de novo* collagen production (27, 29). High concentrations in BALF of collagen degradation products, such as Hyp, could be interpreted as an indicator of the turnover rate of COL1 in the extracellular matrix and of the active fibrotic burden in the tissue. If these products reach the alveolar space and subsequently general circulation, their quantification in saliva could provide indirect information on fibrotic activity in the lung. However, COL1 is also a structural component of the periodontal ligament (139, 140) and its turnover and degradation rate increases in oral diseases such as periodontitis (141). Therefore, to validate these salivary biomarkers as pulmonary indicators, it is necessary to estimate the local oral contribution by establishing differentiated stratification criteria for patients with active oral pathology versus subjects with periodontal health.

Currently, intact COL1 protein is not usually quantified directly; instead, the fibrotic burden is estimated from its degradation products (141) or by quantifying the expression of the COL1A1 gene in lung tissue or in isolated BALF cells (142, 143). On the other hand, Hyp has been directly quantified in oral fluids (144). Its high structural abundance in the collagen triple helix (29) allows this modified amino acid to be used as an indirect and reliable measure of total tissue collagen biomass.

However, although Hyp concentration accurately reflects extracellular matrix remodeling and provides clues to fibrotic activity, it is not a metabolite exclusive to the pulmonary interstitium. Its distribution is ubiquitous in all connective tissues. While it is quantifiable in saliva (144), it cannot be used in isolation as a systemic marker of pulmonary fibrosis, since local oral production would act as a critical confounding factor. In inflammatory pathologies that affect the oral connective tissue, such as gingivitis or periodontitis, the ECM is subjected to accelerated turnover, which significantly increases the salivary concentration of collagen degradation products and, specifically, of free Hyp (141, 144).

Fibronectin is another matrix component that plays a key role in the progression of various lung diseases. In the case of IPF, the overexpression and subsequent excessive accumulation of fibronectin lead to a series of changes that result in increased stiffness and reduced distensibility causing loss of lung tissue functionality (74). In COPD and asthma, fibronectin contributes to bronchial wall thickening and airflow obstruction (145). Likewise, during acute respiratory infections and acute respiratory distress syndrome (ARDS), fibronectin levels may increase in response to epithelial injury, reflecting the activation of repair mechanisms and increased permeability of the alveolar-capillary barrier (146, 147). Fibronectin levels can be measured in BALF by ELISA or proteomic analysis as a marker of ECM remodeling in IPF and other ILDs (6, 25, 74, 87–90) and potentially in saliva by ELISA in inflammatory respiratory conditions (148).

Alterations in the expression and activity levels of elastases have been associated with COPD (45) as an imbalance between proteolytic enzymes, such as neutrophil elastase and their natural inhibitors—primarily α1-antitrypsin—promotes excessive degradation of elastin in the extracellular matrix. This loss of structural integrity of elastic fibers leads to a reduction in the recoil function of the pulmonary parenchyma. In turn, it causes alveolar destruction, hyperinflation and a progressive decline in respiratory capacity. Other factors such as oxidative stress, aging and exposure to toxic agents, including tobacco smoke, initiate inflammatory processes that become self-perpetuating. This, in turn, can cause persistent injury and damage to the lung, accelerating elastin fragmentation and degradation. Consequently, inflammatory and pulmonary remodeling processes are prolonged and contribute to chronic respiratory diseases (26, 149). Elastase activity can be measured in BALF using enzymatic assays (123) and in the saliva of COPD patients using validated ELISA kits. This activity correlates with disease exacerbations and smoking (150). Elastin degradation products can also be quantified in saliva samples by mass spectrometry (87, 125).

#### 4.1.2 Markers of epithelial damage: KL-6

Elevated levels of KL-6 in blood are due to the release of these antigens following disruption of the alveolar–capillary barrier, where alveolar concentrations of MUC1 greatly exceed basal production in AECII. KL-6 is a characteristic antigen of MUC1, but it is not exclusive to alveolar cells, as it has also been identified in other glandular tissues capable of secreting MUC1, such as salivary glands and saliva (138). In such a way KL-6 levels in saliva may reflect not just lung but also oral alterations.

Since the characterization of KL-6 by Kohno et al. (47), it has been observed that antigen levels in BALF and serum correlate with each other. Since then, it has been hypothesized that this positive correlation between both biological fluids may serve as a direct indicator of the degree of alveolar epithelial damage or regeneration (24, 47). Interestingly, recent reviews have compiled cases in which systemic biomarker levels show correlating values between serum and saliva (15). Therefore, this would suggest that saliva could serve as an alternative biological fluid to serum for measuring KL-6 and consequently, the severity of alveolar epithelial damage. However, in order for saliva to be used as an alternative diagnostic fluid, it would be necessary to establish a baseline reference value of KL-6 in saliva, for which specific studies will be required (47, 138).

#### 4.1.3 Remodeling markers: matrix metalloproteinases

Matrix metalloproteinases are enzymes involved in the degradation and restructuring of ECM components. They are essential for maintaining the balance between matrix degradation and synthesis. When fibrotic processes develop in the lung, there is an increase in the expression of MMPs. In these cases, inappropriate regulation or overexpression of certain MMPs has been detected, mainly MMP-1, MMP-2, MMP-7, MMP-9, and MMP-12 (53, 119). These enzymes cause excessive degradation of the extracellular matrix and the release of bioactive fragments that act as profibrotic and proinflammatory signals (40). These signals stimulate the activation of myofibroblasts and fibroblasts, resulting in the accumulation of fibrotic tissue and the loss of normal alveolar structure in a vice circle.

In the case of COPD, the overexpression of MMP-9 and MMP-12 by macrophages and neutrophils has been directly associated with the destruction of the alveolar parenchyma and the development of emphysema. In contrast, a more prominent contribution of MMP-2 and MMP-7 is observed in ILDs, related to cell migration, interstitial remodeling and fibrotic progression. Ji et al. (151) described a negative correlation between salivary MMP-9 and pulmonary function in patients with COPD, although it did not allow discrimination between smokers and non-smokers.

**FIGURE 3:**
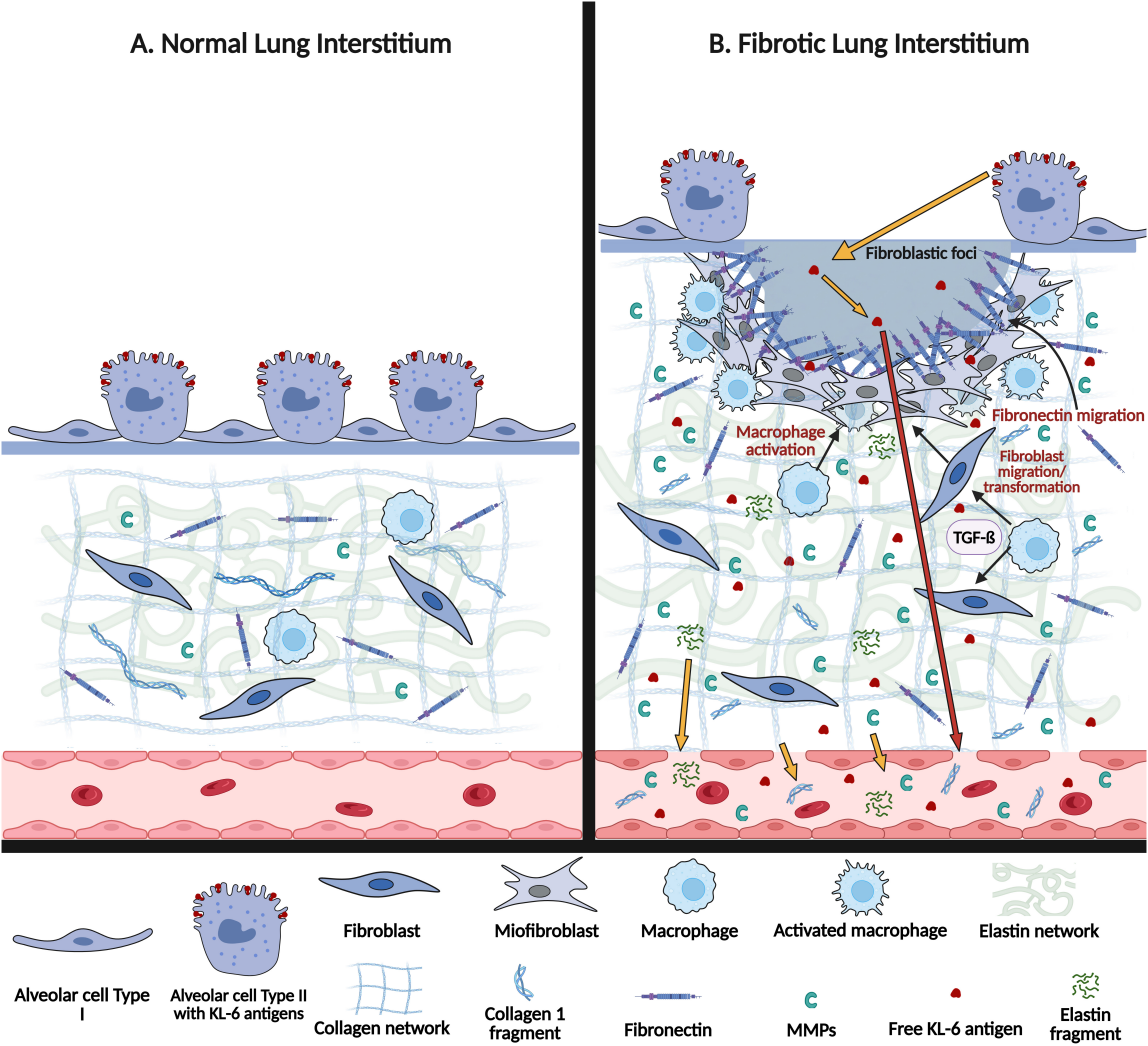
Schematic representation of normal **(A)** versus fibrotic **(B)** lung interstitium leaking metabolic markers detectable in blood and other fluids as saliva. In the healthy lung **(A)**, the alveolar epithelium remains intact, preserving barrier integrity and maintaining a balanced extracellular matrix (ECM) composed of type I collagen (COL1), fibronectin and elastin fibers organized in a three-dimensional network. This structure supports alveolar stability, allowing proper expansion during inspiration and elastic recoil during expiration, thereby ensuring efficient gas exchange and normal lung compliance. In contrast, the fibrotic lung (B) is characterized by alveolar epithelial injury and increased permeability. The activation of alveolar macrophages and TGF-β increased secretion promote fibroblast recruitment, proliferation, and differentiation into myofibroblasts. These processes lead to the formation of fibroblastic foci and excessive deposition of ECM components, including COL1 and fibronectin. These changes thicken and harden the interstitium, reducing the diffusion of respiratory gases. Fibrotic remodeling is further exacerbated by dysregulated proteolytic activity, particularly mediated by matrix metalloproteinases (MMPs) and other elastolytic enzymes derived from inflammatory cells as macrophages. MMPs degrade elastin fibers into bioactive elastin fragments and free detectable specific amino acids as desmosine and isodesmosine. Persistent inflammation with macrophage activation and abnormal ECM accumulation together promote the increase of molecules involved in the fibrotic process and the leakage of several (COL1, Hyp, KL6, MMPs, elastin fragments and desmosine,…) to the circulation serving as detectable molecular markers of the fibrotic progression in body fluids as blood, BALF and saliva. Created in BioRender by Angel-Reimundez et al. (2026) (https://biorender.com/b17o4zh).

In IPF, MMP-7 is consistently overexpressed in the alveolar epithelium and in peripheral blood, what has been associated with cell migration processes, interstitial remodeling and fibrotic progression of parenchyma (39, 152). In turn, Pardo and Selman (39) reviewed how MMP-2 is overexpressed in IPF lung tissue and how it was detected as increased in BALF, highlighting its role in monitoring basement membrane degradation and fibroblast migration during matrix remodeling in pulmonary fibrosis (39, 40) (see Figure 3). By analogy, the detection of elevated MMPs in saliva of IPF patients could be associated with active remodeling phases, although there is still no specific evidence. In fact, there is little evidence on MMP levels in saliva and their variations, which is mandatory to establish the interest of saliva in the early clinical management of IPF by serving as a non-invasive tool (123, 153). However, for these applications to be implemented in hospital practice, it is crucial to establish reference ranges that are currently undefined (15).

Currently, none of these molecules is used to diagnostic or monitoring IPF disease despite their clear feasibility, as previous studies have strongly linked them to different biological processes underlying the pathophysiology of IPFs. This biomarkers can report on epithelial integrity, fiber deposition, ECM turnover and local inflammation in a differentiated way, and they can already be detected in BALF and saliva. This does not rule out other candidates which could meet the criteria in the near future.

## 5 Highlighted issues and future studies

The current diagnosis of interstitial lung diseases (ILDs) is based primarily on three complementary clinical pillars: high-resolution computed tomography (HRCT), histological evaluation (when available) and pulmonary function tests, particularly DLCO (1, 2, 7, 21). HRCT remains the most important imaging tool, allowing for the identification of characteristic fibrotic patterns and estimation of disease extent. Histology, on the other hand, provides direct tissue-level confirmation of architectural distortion, inflammatory infiltration, and extracellular matrix remodeling (21), but requires a biopsy sample, which involves a certain risk. Pulmonary function tests are essential for assessing the physiological consequences of fibrosis and for monitoring the disease, although they do not report on the molecular mechanisms underlying disease activity. In this context, the quantitative approach to biomarkers from BALF and/or saliva samples would provide additional information to help in early diagnosis and follow-up, which should be understood as a complementary tool that has been ignored until now. Molecular biomarkers would provide a more complete understanding of epithelial injury, matrix turnover, and inflammatory activation that are not obtained with standard clinical methods. Compared to the conventional approach, BALF offers the advantage of sampling directly from the alveolar and bronchiolar microenvironment, making it particularly suitable for the study of molecules released at the site of injury (6, 70, 87). The humoral component of BALF is now ruled out and only the cell fraction is studied by cytometric analysis. On the other hand, saliva, although less specific, provides non-invasive and repeatable sampling that may be useful for screening and follow-up if its correlation with BALF is validated (15, 72, 96). Therefore, BALF and saliva-based biomarkers can help refine early detection, phenotyping, prognostic stratification and treatment follow-up. In parallel, emerging omics and computational platforms, such as AI-based technologies, can accelerate biomarker discovery and support the integration of clinical, radiological, functional and molecular data into more robust diagnostic and predictive models.

Despite the biomarkers described in scientific literature have been characterized relatively extensively in BALF, and to a lesser extent in saliva, the lack of studies simultaneously analyzing both fluids in the same patient cohorts limits the translational interpretation of current findings (15, 47).

Before, research should prioritize the design of prospective studies comparing levels of fibrotic biomarkers of lung injury and matrix remodeling in BALF and saliva. These include those mentioned in this review: COL1, Hyp, fibronectin, elastin, KL-6, and MMPs. Furthermore, other molecules emerge as potential to be included in this framework, such as CFHR1, CRTAC1, CXX3CL1, CD44 or periostin. In addition, their association with the clinical, functional and radiological progression of IPFs needs to be investigated (24). Currently, there is no specific evidence demonstrating a quantitative, reproducible and clinically validated correlation between fibrotic biomarker levels in BALF and saliva related to major pulmonary and obstructive diseases. Likewise, these biomarkers should be integrated with pulmonary function testing and HRCT to define robust correlations and the true prognostic–predictive value of saliva (15, 154).

The mechanisms by which extracellular matrix macromolecules pass from the alveolar compartment to the systemic circulation and from there to saliva have been proposed mainly on a theoretical base. These mechanisms are based on disruption of the alveolar–capillary barrier, increased vascular permeability and changes in lymphatic drainage (94, 120, 155, 156). However, these processes have not been quantitatively characterized or compared across different pulmonary diseases (fibrotic interstitial lung diseases, COPD, sarcoidosis or hypersensitivity pneumonitis), which limits the ability to establish pathophysiological models that robustly explain the relationship between BALF and salivary values. Therefore, the development of comparative studies among IPFs subtypes would be useful to identify disease-specific salivary patterns or signatures that allow discrimination between phenotypes with predominant inflammation versus predominant fibrosis.

It is also necessary that a standard protocol for saliva collection and processing will be established in the design of these prospective studies. Variability in collection timing, fasting status, storage conditions or the addition of protease inhibitors can substantially affect biomarker quantification (138). Therefore, for saliva to be clinically useful, standardization and validation of protocols in the context of pulmonary disease are required, as they have previously been highlighted in other saliva-based diagnostic settings (131, 157).

Another limitation of saliva is the biological specificity of salivary biomarkers, since molecules such as MMPs, fibronectin or COL1 may be elevated due to local diseases in the oral cavity (e.g., periodontitis) and they may not necessarily reflect pulmonary involvement (138, 157). In future applications, periodontal status and oral health should be controlled in a standardized protocol-driven manner to explore more specific biomarkers that clearly reflect pulmonary status.

In this context, future studies should focus on two main lines of research. First, longitudinal trials which analyze the proposed biomarkers in relation to serial changes in lung function and their response to antifibrotic therapies. The aim will be to assess their usefulness for monitoring and treatment, as well as for the early detection of pulmonary exacerbations. Second, studies that systematically characterize salivary “background noise” to quantify the local contribution of the salivary glands, periodontal disease, smoking and oral infections relative to biomarker levels. In this way, it will be possible to separate the lung-derived signal from other external sources.

Finally, it is important to note that the development of saliva-based point-of-care tools could facilitate non-invasive monitoring of disease progression and treatment response in patients with IPFs and other pulmonary diseases. To this end, it will be necessary to leverage immunochemical and proteomic platforms that have already been applied in other salivary diagnostic contexts (33, 95, 96, 124, 157). Devices capable of rapidly and consistently quantifying panels of fibrotic and inflammatory biomarkers in saliva will need to be designed. The aim will be to integrate these results into clinical decision-making algorithms that define in which scenarios saliva could replace or extend the intervals between the use of BALF for follow-up, progression screening and therapeutic monitoring. This approach will also make it possible to determine in which cases BALF will remain indispensable for diagnostic phenotyping or the selection of targeted therapies.

## 6 Conclusion

BALF is the reference matrix for studying *in vivo* the cellular and molecular mechanisms of many pulmonary diseases, particularly IPF. It directly reflects the alveolar microenvironment, including inflammation, fibrosis, infection and neoplasia. The combination of histological techniques, molecular techniques (qPCR, RT-qPCR, FISH, NGS) and immunoassays allows for a highly complete characterization of fibrotic burden, epithelial injury and extracellular matrix remodeling in these diseases. The molecular biomarkers—collagen, hydroxyproline, fibronectin, elastin, KL-6 and MMPs—may provide a physiopathologically relevant panel for the detection and follow-up of IPF. These biomarkers cover different phases of the fibrotic and remodeling process, from epithelial damage to the deposition and degradation of the extracellular matrix. BALF data indicate that their levels are associated with disease activity and deterioration of pulmonary function, supporting their potential value as prognostic and/or predictive markers.

On the other hand, saliva emerges as a promising matrix for monitoring biomarkers of pulmonary diseases, due to its minimally invasive collection, the feasibility of serial sampling and the availability of immunochemical, proteomic and molecular platforms already adapted to this fluid. The fact that saliva shares a relevant fraction of the plasma proteome and has demonstrated diagnostic utility in infectious, oncologic, and systemic contexts supports its exploration as a partial substitute for, or complement to, BALF in fibrotic pulmonary diseases. The hypothesis that damage to the alveolar–capillary barrier and increased vascular permeability would allow the passage of matrix macromolecules, and that epithelial markers can go from the alveolus into the bloodstream and subsequently into saliva represents a potential area of investigation to determine quantitative correlations between both fluids. Thus, saliva could be used to estimate active fibrotic burden (Hyp/COL1), epithelial injury (KL-6) and matrix remodeling (MMPs). Thereby facilitating the clinical follow-up of IPFs without the need for repeated invasive bronchoscopy procedures. The main current limitation is the lack of studies that simultaneously measure these biomarkers in BALF and saliva using the same cohorts, techniques and conditions. Therefore, a robust and clinically validated quantitative correlation between these two matrices in IPF has not yet been established.
